# Physicochemical properties of polysaccharides from *Dendrobium officinale* by fractional precipitation and their preliminary antioxidant and anti-HepG2 cells activities in vitro

**DOI:** 10.1186/s13065-018-0468-4

**Published:** 2018-09-25

**Authors:** Shangping Xing, Xiaofeng Zhang, Hannu Ke, Ji Lin, Yuechun Huang, Gang Wei

**Affiliations:** 1 School of Pharmaceutical Science, Guangzhou University of Chinese Medicine, Guangzhou, 510006 China; 2 The First Affiliated Hospital of Guangzhou University of Chinese Medicine, Guangzhou, 510006 China

**Keywords:** *Dendrobium officinale*, Polysaccharide fractions, Fractional precipitation, Antioxidant activity, HepG2 cells

## Abstract

**Background:**

*Dendrobium officinale* as a precious traditional Chinese herb is widely used in medicines and health supplements. Thus the extraction, purification and biological activities of polysaccharides from the stem of *Dendrobium officinale* have significant meaning on theory and application value.

**Methods:**

The crude *Dendrobium officinale* polysaccharide (DOP) was obtained by hot water extraction- ethanol precipitation method, and four new polysaccharide fractions (DOP-40, DOP-50, DOP-60, and DOP-70) were further obtained from the crude DOP by fractional precipitation with ethanol method, then four fractions were further purified by Toyopearl-H65F gel resin. The molecular weight and monosaccharide composition of four purified fractions were determined by high performance anion exchange chromatography and high performance liquid chromatography. The antioxidant activities of them were evaluated by the reducing power assay, and the superoxide anion, 2,2-diphenyl-1-picrylhydrazyl (DPPH), and hydroxyl free radicals scavenging assays, respectively. Finally, the anticancer activities of them were investigated via the MTT assay and the western blot analysis using HepG2 cells.

**Results:**

Among these four purified fractions were mainly composed of d-mannose and d-glucose with different molar ratios, and their average molecular weights were 999, 657, 243 and 50.3 kDa, respectively. What’s more, DOP-70 always exhibited the strongest antioxidant and anticancer activities, while DOP-40 and DOP-60 showed very close antioxidant and anticancer activities which were better than that of DOP-50. The western blotting analysis also showed that DOP-40, DOP-60, and DOP-70 induced apoptosis in HepG2 human liver cancer cells through the Bcl-2 and Bax-dependent pathway.

**Conclusions:**

Fractional precipitation with ethanol could successfully apply to extract four new polysaccharide fractions from *Dendrobium officinale* stems, and the polysaccharide fractions possessed efficient antioxidant and anticancer activities, especially DOP-70.

## Background

*Dendrobium officinale*, a perennial epiphytic herb in the *Orchidaceae* family, is the most precious species in *Dendrobium* genus found in Zhejiang, Anhui, Yunnan and Guangxi provinces of China [1, 2]. Its stem has been traditionally consumed as both food and medicine recorded in “Pharmacopoeia of the People’s Republic of China”, and it has been proved to possess enhancing immunity, anti-inflammatory, antioxidant and anticancer activities [3–5]. In recent years, increasing evidence proved its potentially significant functions and its slow growth rate, which resulted in a sharp rise in its price and more studies on its chemical constituents and biological activities [6, 7].

Recently, extensive studies have been demonstrated that polysaccharides from *Dendrobium officinale* stems possessed could protect RAW 264.7 cells against oxidative injury [8], inhibit TNF-a-induced apoptosis in A-253 cell line [9], and enhance intestinal mucosal immune activity [10]. To date, fractional precipitation with ethanol has already become a rapid, feasible and reproducible way to extract initial purify of aqueous extracts [11]. In addition, different concentrations of ethanol have been reported to generate corresponding protein content, molecular weight, monosaccharide composition and bioactivity of the products [12]. However, the physicochemical properties and functions of polysaccharide fractions from *Dendrobium officinale* stems extracted by ethanol fractional precipitation method still remained unknown. In this study, four new polysaccharide fractions were obtained via fractional precipitation, and their bioactivities were further evaluated with the purpose of providing novel natural antioxidant and anticancer compounds from *Dendrobium officinale* stems.

## Experimental

### Plant material

**Fig. 1:**
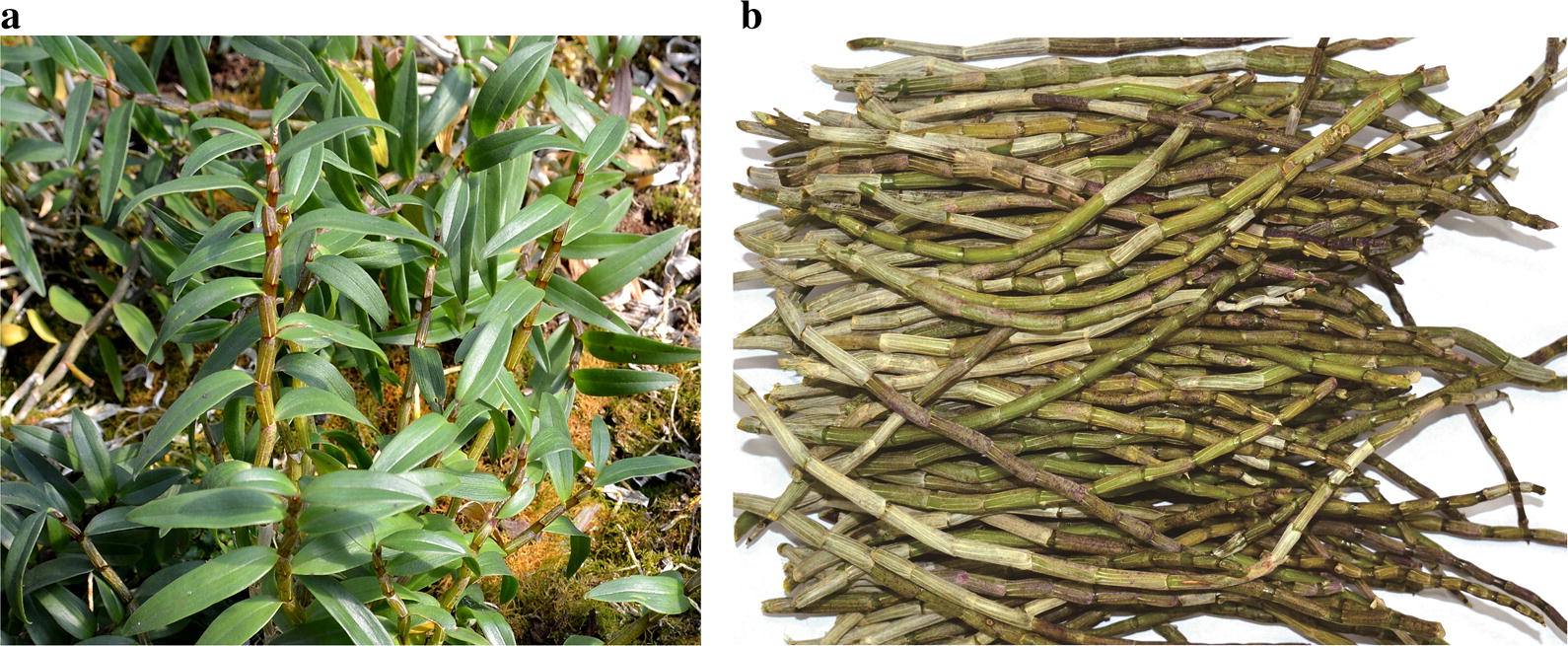
Images of *Dendrobium officinale* (**a**), and its stems (**b**)

*Dendrobium officinale* were collected from the Zhejiang Province in China (Fig. 1), as we described previously [8]. The voucher specimens were preserved in the School of Pharmaceutical Science. The stems of *Dendrobium officinale* were dried up at 60 °C and ground into fine powder for the subsequent studies.

### Cell culture

The human liver cancer HepG2 cells were obtained from Shanghai Cell Bank (Shanghai, China). HepG2 cells cultured in DMEM medium (10% (v/v) FBS, 100 mg/ml streptomycin and 100 U/ml penicillin) and incubated at 37 °C with 5% CO_2_.

### Reagents

Toyopearl-H65F (TOSH, Japan); Coomassie brilliant blue G-250, Ascorbic acid, and Bovine serum albumin (Aladdin, USA); Tris-(hydroxymethyl)-aminomethane (Tris), Ethylenediaminetetraacetic acid (EDTA), and Salicylic acid (Sinopharm Chemical Reagent Co., Ltd, China); 2,2-diphenyl-1-picrylhydrazyl (DPPH), Dextrans, and MTT [3-(4,5-dimethylthiazol-2-yl)-2,5-diphenyl tetrazolium bromide] (Sigma-Aldrich, USA); d-mannose, l-rhamnose, d-galactose, d-glucose, and d-arabinose (Chinese Materials Research Center, China); Antibodies against β-actin, Bax, Bcl-2, goat anti-rabbit IgG, and goat anti-mouse IgG (Cell Signaling Technology, USA). All other reagents used were of analytical grade.

### Extraction, fractionation, and purification of polysaccharide

*Dendrobium officinale* dry powders were refluxed with petroleum ether at 70 °C for 2 h, then with 20 volumes of 80% ethanol for 2 h at 90 °C to remove polar constituents and filtered. The residue was extracted twice with 45 volumes of distilled water at 100 °C for 2 h, then the aqueous was concentrated under vacuum and added with 4 volumes of 80% ethanol at 4 °C overnight, and centrifuged at 5000 rpm for 10 min. Subsequently, the precipitate was dissolved in distilled water and added with the Sevage reagent to remove protein present. The solution was precipitated with 80% ethanol and then lyophilized to obtain crude *Dendrobium officinale* polysaccharide (DOP) [13, 14].

**Fig. 2:**
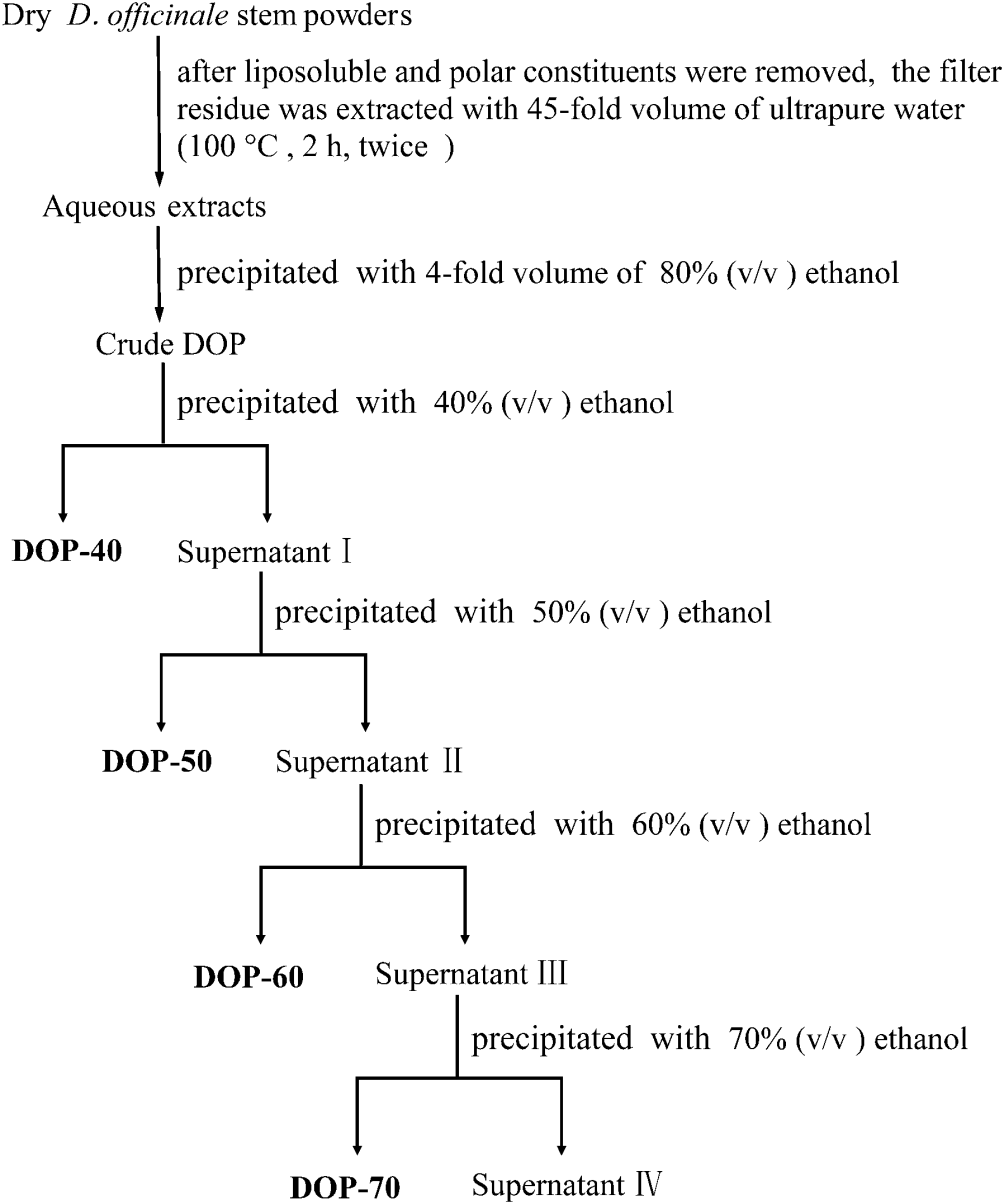
Extraction and fractionation procedure of polysaccharide fractions from *Dendrobium officinale* stems

The DOP (2 g) was dissolved in 100 ml distilled water, then fractional precipitation with ethanol was applied to separate the solution [15], followed by different volumes of anhydrous ethanol (66.67 ml, 33.33 ml, 50 ml and 83.33 ml) were added to the solution to create a series of final concentration (40%, 50%, 60% and 70%, v/v) of ethanol solution, successively. After the mixture was stored at 4 °C overnight, the four precipitates (DOP-40, DOP-50, DOP-60 and DOP-70) were collected by centrifugation and then freeze-dried, respectively (Fig. 2).

### Purification of polysaccharide fractions

The four polysaccharide fractions were further purified by Toyopearl-H65F gel resin (1.6 × 80 cm) and eluted with 0.2 mol/l NaCl at a flow rate of 0.5 ml/min [16, 17]. Then the solution was collected in accordance with four elution peaks (DOP-40, DOP-50, DOP-60, and DOP-70), concentrated, and dialyzed against distilled water for 48 h, fresh distilled water was replaced every 4 h, respectively. Finally, four dialyzed polysaccharide fractions solution were lyophilized for further study. The UV spectrophotometry was used to measure the OD value at 490 nm.

### Determination of neutral sugar and protein contents

The neutral sugar and protein contents of DOP-40, DOP-50, DOP-60, and DOP-70 were determined respectively by the improved phenol–sulfuric acid method taking glucose as the standard sample [18], and the Coomassie blue staining (G-250) assay taking BSA as the standard sample [19]. The experiment was repeated in triplicate (n = 5 each time).

### Molecular weight determination

The average molecular weights of DOP-40, DOP-50, DOP-60, and DOP-70 were determined by high performance gel permeation chromatography (HPGPC) using a TSK GMP_WXL_ chromatographic column (7.8 × 300 mm, 2 μm, TOSH, Japan). The mobile phase was 20 mM CH_3_COONH_4_ with a flow rate of 0.8 ml/min, and the standard curve was established using T-series dextran (MW: 1270, 5220, 11600, 48600, 80900, 273000, and 409800 Da) [20, 21].

### Monosaccharide composition analysis

The monosaccharide compositions of DOP-40, DOP-50, DOP-60, and DOP-70 were analyzed by HPLC equipped with a Kromasil 100-5C18 chromatographic column (4.6 × 250 nm, 5 μm, AKZO NOBEL, Sweden) [22]. The compositions of mobile phase were acetonitrile (solvent A) and 0.1 M aqueous KH_2_PO_4_ acetonitrile (solvent B), the flow rate was 0.8 ml/min, and the wavelength of detection was 250 nm. Five monosaccharide standards (d-mannose, l-rhamnose, d-galactose, d-glucose, and d-arabinose) were used to establish standard curves, and the monosaccharide compositions and molar ratio of DOP-40, DOP-50, DOP-60, and DOP-70 were determined.

### Determination of antioxidant activities

#### Reducing power assay

The polysaccharide fractions (DOP-40, DOP-50, DOP-60, and DOP-70) were prepared into concentrations of 0.5 mg/ml, 1 mg/ml, 2 mg/ml, 3 mg/ml and 5 mg/ml with distilled water for antioxidant activities assays, ascorbic acid was used as the positive control, and each assay was repeated three times.

The reducing power of sample was determined according to the method with some modifications [23]. The system contained 2 ml of K_3_Fe(CN)_6_ (1%, w/v), 2 ml of sample solution, and 2 ml of phosphate buffer (0.2 mol/l, pH 6.6). After the mixed solution was incubated at 50 ^◦^C for 20 min, 2.5 ml of trichloroacetic acid (10%, w/v) was added to the solution to stop the reaction, then the solution was centrifuged at 3000 rpm for 15 min, after 2 ml of the supernatant was mixed with 2 ml of distilled water and 1 ml of ferric chloride (0.1%, w/v) for 10 min at room temperature. The absorbance representing the ability of the reducing power was measured at 700 nm.

#### Superoxide anion radical (O_2_^−^) scavenging assay

O_2_^−^ scavenging activities of DOP-40, DOP-50, DOP-60, and DOP-70 were evaluated by pyrogallol autoxidation method, with slight innovation [24]. Four sample solutions were diluted with Tris–HCl buffer (0.05 mol/l, pH 7.4) to form various mixed solutions (0.5 mg/ml, 1 mg/ml, 2 mg/ml, 3 mg/ml, and 5 mg/ml), and then 0.4 ml of pyrogallic acid (60 mM) was added to the mixture solutions at 25 °C. The final mixture was shaken vigorously and incubated at 25 °C for 5 mines, and the absorbance of the reaction mixture was determined at 325 nm. The O_2_^−^ scavenging rate was calculated according to the following formula:

1 $${\text{O}}_{2}^{-}\,{\text{scavenging rate }}\left( {\%} \right) = \left[ {1 - \frac{{{\text{A}}_{1} }}{{{\text{A}}_{0}}}} \right] \times 100$$

where A_1_ is the absorbance of sample, and A_0_ is the absorbance of blank (distilled water instead of sample solution).

#### DPPH radical scavenging assay

DPPH radical scavenging activities of DOP-40, DOP-50, DOP-60, and DOP-70 were determined according to a reported method with a few modifications [25]. DPPH powder was dissolved in anhydrous ethanol (40 mg/ml) as the stock solution. Then aliquots of 2 ml of polysaccharide fraction samples solution were added to 2 ml of DPPH stock solution in a cuvette and incubated for 20 min in the dark, and the absorption was determined at 517 nm. The DPPH radical scavenging rate of polysaccharide fractions was expressed as the following equation:

2 $${\text{DPPH radical scavenging rate }}\left( {\% } \right) = \left[ {1 - \frac{{ {\text{A}}_{i} - {\text{A}}_{j} }}{{ {\text{A}}_{c} }}} \right] \times 100$$

where A_*i*_ is the absorbance of the solution including DPPH and sample as the experimental group, A_*j*_ is the absorbance of the solution which contains DPPH and anhydrous ethanol as the background group, A_*c*_ is the absorbance of the anhydrous ethanol solution as the blank group.

#### Hydroxyl radical scavenging assay

Hydroxyl radical scavenging activities of DOP-40, DOP-50, DOP-60, and DOP-70 were performed with a reported method described [26]. Briefly, 2 ml of Fe_2_SO_4_ (6 mM), 2 ml of salicylic acid–ethanol (6 mM) and 2 ml of the four polysaccharide fractions were mixed thoroughly in the cuvette. After 10 min, 2 ml of the H_2_O_2_ (6 mM) was added to the mixture solution, the reaction solutions were incubated in 37 °C thermostat water bath for 30 min. The absorbance of solutions was measured at 510 nm. The results were calculated by the following equation:

3 $${\text{Hydroxyl radical scavenging rate }}\left( {\%} \right) = \left[ {1 - \frac{{{\text{A}}_{1} - {\text{A}}_{2} }}{{ {\text{A}}_{0} }}} \right] \times 100$$

where A_1_ is the absorbance of sample solution as the experimental group, A_2_ is the absorbance of the mixture solution which distilled water instead of H_2_O_2_ as the background group, A_0_ is the absorbance of the mixture solution which distilled water instead of the sample as the blank group.

### Anticancer activity assay in HepG2 cells

#### Influence of polysaccharide fractions on HepG2 cell viability

The anticancer effect of polysaccharide fractions were examined by MTT assay. HepG2 cells (5 × 10^3^ cells/well) were seeded in 96-well plates for 24 h, then treated with DOP-40, DOP-50, DOP-60, and DOP-70 (25 mg/ml, 50 mg/ml, 100 mg/ml, 200 mg/ml, and 400 mg/ml) for 24 h and 48 h. After adding 100 μl of MTT reagent (0.5 mg/ml) for 4 h incubation, the medium was replaced with 150 ml DMSO to dissolve the formazan. The absorbance value of the experimental group (A_experimental_) and control group (A_control_) were read at 490 nm by Victor X3 (PerkinElmer, USA) and inhibition rate was calculated as follows:

4 $${\text{Inhibition rate}}\left( {\% } \right) = \left( {1 - \frac{{{\text{A}}_{\text{experimental}} }}{{{\text{A}}_{\text{control}} }}} \right) \times 100$$

### Western blot analysis

HepG2 cells (2.5 × 10^5^ cells/well) were seeded in 6-well plates, and treated with 400 mg/ml DOP-40, DOP-50, DOP-60, and DOP-70 for 48 h, respectively. The protein was lysed and harvested by RIPA buffer (1% PMSF). Then according to the standard western blot protocol, the samples were incubated with the primary antibodies (β-actin, Bax and Bcl) for overnight at 4 °C. After incubated with the corresponding HRP-labeled secondary antibody (Goat anti-mouse IgG or anti-rabbit IgG) at room temperature for 1 h. The hybridization signal of the protein band was examined using ECL detection reagents.

### Statistical analysis

All the experimental data were statistically analyzed with SPSS 19.0 software and expressed as mean ± standard deviation (SD) by GraphPad Prism 6.0 software. *p < 0.05 and **p < 0.01 are determined as significance.

## Results

### Extraction and purification of polysaccharide fractions

**Fig. 3:**
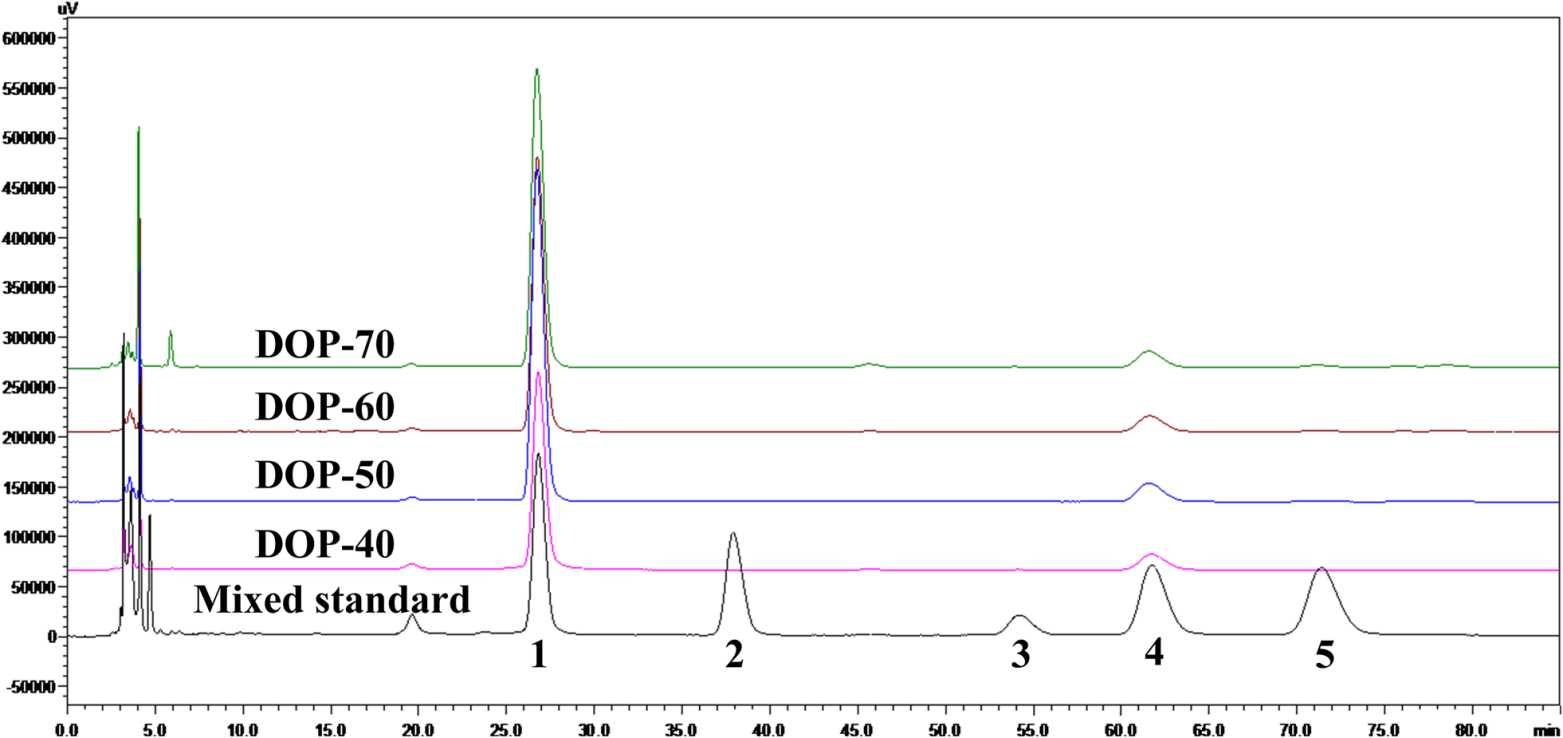
Distilled NaCl elution curves of DOP-40 (**a**), DOP-50 (**b**), DOP-60 (**c**), and DOP-70 (**d**)

Four new *Dendrobium officinale* polysaccharides (DOP-40, DOP-50, DOP-60, and DOP-70) were successively obtained from 10 mg/ml crude DOP solution by fractional precipitation with final concentrations (40%, 50%, 60% and 70%, v/v) of ethanol solution (Fig. 2), and further purified by Toyopearl-H65F column chromatography. As the results showed (Fig. 3a–d), elution curves of four fractions produced symmetrical single elution peak eluted with 0.2 mol/l NaCl solutions. The yields of DOP-40, DOP-50, DOP-60, and DOP-70 from the crude DOP were 59.0%, 23.8%, 7.5%, and 5.0%, respectively.

### Physicochemical properties analysis

The physicochemical properties of DOP-40, DOP-50, DOP-60, and DOP-70 from *Dendrobium officinale* stems were listed in Table 1. The protein content of each fraction were 1.68%, 1.12%, 0.03%, and 0.34%, the neutral sugar content of each fraction were 8.32%, 10.43%, 21.33%, and 6.82%, respectively. Among them, DOP-70 contained the lowest content of protein, and the highest content of neutral sugar.

**Table 1:** The yield and physicochemical properties of polysaccharide fractions from *Dendrobium officinale* stems

Samples	DOP-40	DOP-50	DOP-60	DOP-70
Yield (wt%)	59.31 ± 0.21	23.82 ± 0.23	7.55 ± 0.31	5.25 ± 0.09
Neutral sugar content (wt%)	18.38 ± 1.51	28.38 ± 1.53	13.67 ± 2.78	9.38 ± 0.71
Protein content (wt%)	4.28 ± 0.06	1.68 ± 0.09	1.12 ± 0.03	0.34 ± 0.02
Molecular weight (kDa)	999 ± 0.03	657 ± 0.02	243 ± 0.02	51 ± 0.01
Monosaccharide composition				
d-Mannose/d-Glucose (molar ratio)	6.32 ± 0.15	8.67 ± 0.16	8.34 ± 0.13	8.82 ± 0.05

The molecular weight of polysaccharide was a representative of similar polymer chain length distributed on average. HPGPC was applied to analyze the molecular weight, and the Table 1 indicated that the average molecular weights of DOP-40, DOP-50, DOP-60, and DOP-70 were estimated to be 999 kDa, 657 kDa, 243 kDa and 50.3 kDa, respectively.

**Fig. 4:**
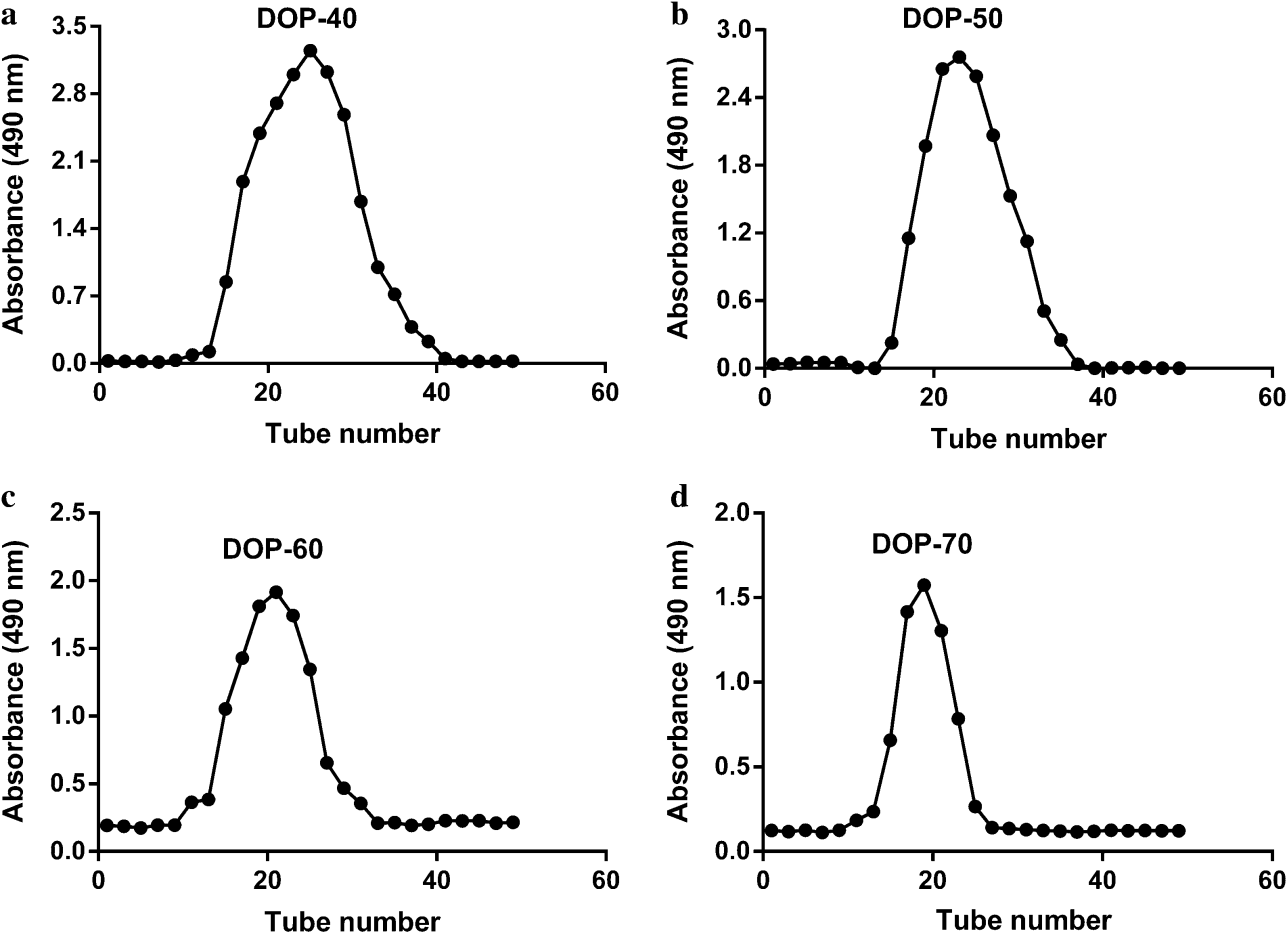
HPLC profile of mixed monosaccharide standards (1-mannose, 2-rhamnose, 3-galactose, 4-glucose, and 5-arabinose), DOP-40, DOP-50, DOP-60, and DOP-70

In this study, the monosaccharide compositions of the four fractions were measured by HPLC, and the results (Fig. 4) were shown that the peaks of all samples were symmetrical and sharp with good sensitivity. According to the mixed standard monosaccharides, four fractions had the same main monosaccharides composition (d-mannose and d-glucose) in different molar ratios of 6.32:1, 8.67:1, 8.34:1, and 8.82:1 respectively. Obviously, abundant d-mannose was found in all the fractions.

### Antioxidant activity in vitro

#### Reducing power

**Fig. 5:**
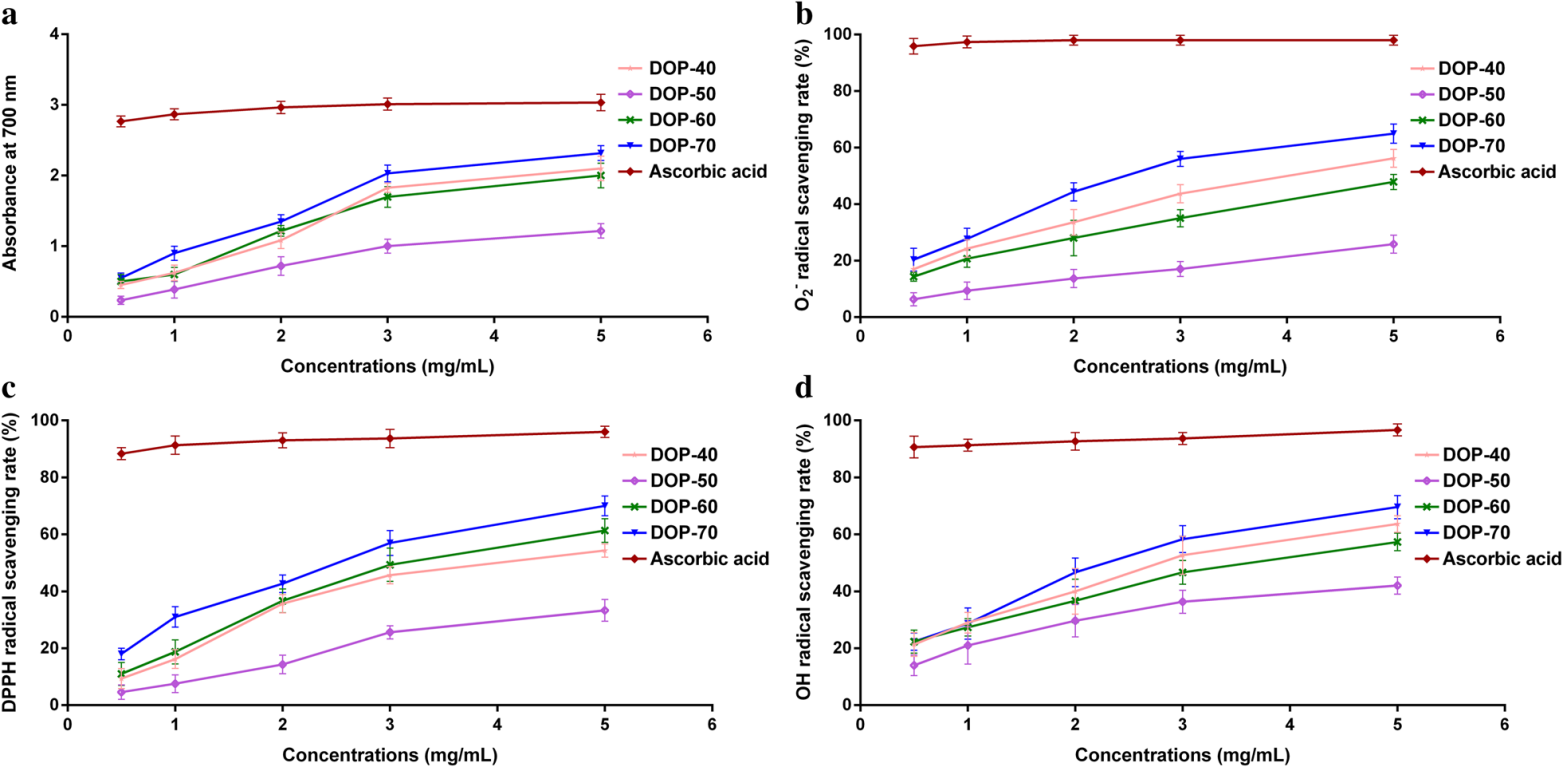
Scavenging activities on the reducing power assay (**a**), superoxide anion radical (**b**), DPPH radical (**c**), and hydroxyl radical (**d**) for DOP-40, DOP-50, DOP-60, and DOP-70

The reducing power of DOP-40, DOP-50, DOP-60, and DOP-70 were measured at 700 nm, and the results were shown in Fig. 5a. The reducing powers of DOP-40, DOP-60, and DOP-70 were very close and entangled from 0.5 to 5 mg/ml, except DOP-50. Among them, DOP-40, DOP-60 and DOP-70 deliberated higher reducing power than DOP-50. Meanwhile, the absorbance of DOP-70 and ascorbic acid were 2.317 and 3.033 at 5 mg/ml, which implied that the reducing power of DOP-70 was 76.4% of that of the positive control (ascorbic acid).

#### Superoxide anion radical (O_2_^−^) scavenging activity

The scavenging abilities of DOP-40, DOP-50, DOP-60, and DOP-70 for O_2_^−^ were shown in Fig. 5b. Obviously the O_2_^−^ scavenging activities of four fractions increased with increasing concentrations, DOP-70 exhibited the higher scavenging ability (IC_50_ = 2625.6 μg/ml) than DOP-40, DOP-50 and DOP-60, but lower than the positive control, and the order was DOP-70 > DOP-40 > DOP-60 > DOP-50. At 5 mg/ml, the O_2_^−^ scavenging rates of DOP-40, DOP-50, DOP-60 and DOP-70 were 56.5%, 25.6%, 47.6%, and 65.3%, respectively.

#### DPPH radical scavenging activity

The results of this study indicated four fractions exhibited strong scavenging activities against DPPH radicals in a dose-dependent pattern (Fig. 5c). At 5 mg/ml, the scavenging rates of DOP-40, DOP-50, DOP-60, and DOP-70 were 55.3%, 32.5%, 61.6%, and 70.3%. DOP-70 showed the strongest effect on DPPH radicals with the lowest IC_50_ value of 2395.8 μg/ml among four fractions, while DOP-50 still exhibited the weakest scavenging capacity. And their IC_50_ decreased in the order of DOP-70 < DOP-60 < DOP-40 < DOP-50.

#### Hydroxyl radical scavenging activity

As illustrated in Fig. 5d, the hydroxyl radical scavenging activities of DOP-40, DOP-50, DOP-60, and DOP-70 increased gradually as the concentration increased from 0.5 to 5 mg/ml. And their scavenging rates increased in the order was: DOP-70 > DOP-40 > DOP-60 > DOP-50, with their IC_50_ values of 2081.7 μg/ml, 2837.7 μg/ml, 3385.3 μg/ml and 5803.8 μg/ml, respectively. At 5 mg/ml of DOP-70, the hydroxyl radical scavenging rate was 69.5% showed the highest effect on hydroxyl radical, while the scavenging rate of DOP-40, DOP-50, and DOP-60 was 63.3%, 42.2%, and 58.6%, respectively. The results were consistent with the results of reducing power, O_2_^−^, and DPPH radical scavenging assays.

### Anticancer activity in HepG2 cells

#### HepG2 cell growth analysis

**Fig. 6:**
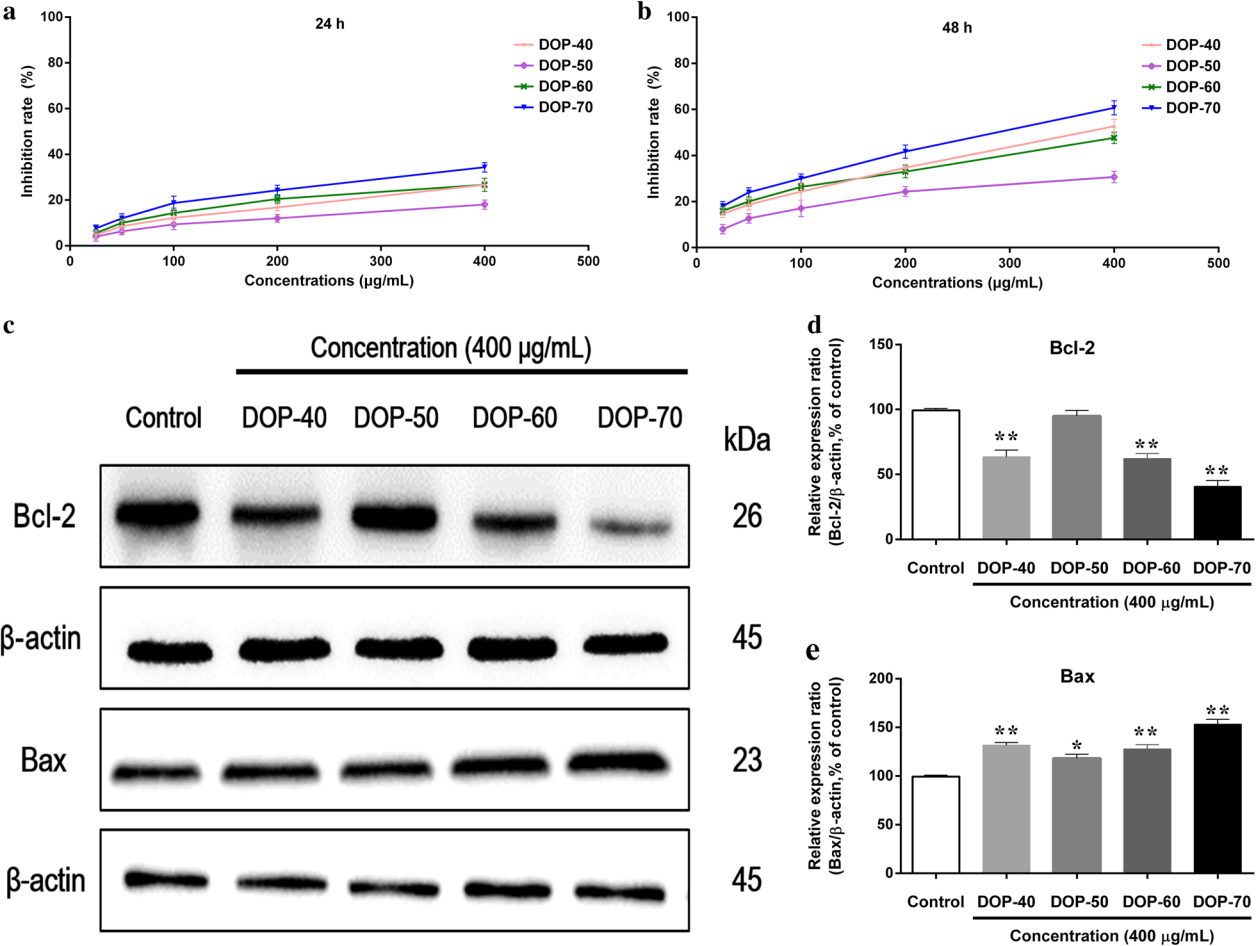
Growth inhibition analysis of HepG2 cells treated with DOP-40, DOP-50, DOP-60, and DOP-70 for 24 h (**a**) and 48 h (**b**) by MTT assay. The protein levels of Bcl-2 (**c**, **e**) and Bax (**c**, **f**) in HepG2 cells treated with DOP-40, DOP-50, DOP-60, and DOP-70 (400 μg/ml) for 48 h were analyzed by western blotting. The bar graphs (mean ± SD) and representative images are shown. *p < 0.05; **p < 0.01 compared with the control group

The effects of DOP-40, DOP-50, DOP-60, and DOP-70 on the growth of the HepG2 cells were first determined by MTT assay. As indicated in Fig. 6a, b, DOP-40, DOP-60, and DOP-70 significantly inhibited the cell proliferation of HepG2 cells in both dose- and time-dependent manner, particularly at a concentration greater than 200 μg/ml at 48 h. In addition, 400 μg/ml DOP-70 markedly increased the inhibition rate by 34.75% at 24 h and 61.36% at 48 h, while the inhibition rates of 400 μg/ml of DOP-40, DOP-50, and DOP-60 were 26.63%, 18.31% and 25.73% at 24 h and 53.56%, 30.36% and 47.38% at 48 h, respectively. So effective concentrations of 400 μg/ml and incubation period of 48 h were used in subsequent experiments.

#### Apoptosis-related protein expressions of Bax, Bcl-2

In order to clarify the mechanisms underlying the inhibition of HepG2 cells by DOP-40, DOP-50, DOP-60, and DOP-70, the protein levels of Bax and Bcl-2 were measured by western blotting. As shown in Fig. 6c–f, the level of anti-apoptotic Bcl-2 was significantly decreased in response to treatment with DOP-40, DOP-60, and DOP-70 at a concentration of 400 μg/ml for 48 h, except DOP-50. Meanwhile, DOP-40, DOP-50, DOP-60, and DOP-70 treatment noticeably increased the pro-apoptotic Bax expression compared to the control group, especially DOP-70. The results showed that DOP-40, DOP-60, and DOP-70 induced apoptosis in HepG2 cells through the Bax- and Bcl-2-dependent pathway.

## Discussion

*Dendrobium officinale* stem contains numerous natural polysaccharides, however different isolation and purification methods have important influence on the molecular weight, monosaccharide composition and bioactivity of *Dendrobium officinale* polysaccharides (DOP). Fractional precipitation with ethanol is a simple and rapid method to purify biologically active polysaccharides from crude plant extracts [27]. In this study, we first extracted the crude DOP with hot water extraction-alcohol precipitation method, then four polysaccharide fractions (DOP-40, DOP-50, DOP-60 and DOP-70) were isolated from the crude DOP via ethanol fractional precipitation method. Importantly, the ethanol concentration is also closely associated with the molecular weight and bioactivity of the polysaccharide fractions. As our results showed, the average molecular weights of DOP-40, DOP-50, DOP-60, and DOP-70, precipitated with final ethanol concentration of 40%, 50%, 60% and 70%, were 999 kDa, 657 kDa, 243 kDa and 50.3 kDa, respectively. Obviously, in the aqueous solution of polysaccharide, the alcohol content is gradually increased by adding ethanol, which can precipitate *Dendrobium officinale* polysaccharide fractions of molecular weight from large to small.

The monosaccharide composition of polysaccharide played a critical role in the biological activity of polysaccharide [28]. HPLC was applied to determine the monosaccharide composition of DOP-40, DOP-50, DOP-60, and DOP-70, and the results indicated that four fractions mainly consisted of d-mannose and d-glucose in different molar ratios. Interestingly, from the HPLC profile analysis, the content of d-mannose was much higher than d-glucose in all fractions.

O_2_^−^ was the first oxygen free radical in the body, and the precursor of other reactive oxygen species, which could lead to cell death, DNA and membrane degradation, and enzyme inactivation [29]. As the O_2_^−^ was toxic, the O_2_^−^ scavenging ability was quite essential for the antioxidant defense in vivo. On the one hand, the reducing power as the important indicator of potential antioxidant activity may directly reflect the reduction of Fe^3+^ to Fe^2+^ by donating an electron [30]. On the other hand, DPPH, as hydrogen donors, owns a characteristic absorption of proton free radical in spectrophotometric method, so the DPPH assay has been extensively used to evaluate antioxidant activity of natural products [31]. Furthermore, the hydroxyl radical was very dangerous compound to the health, which was closely related to several neurological autoimmune diseases, and could damage nucleic acid, amino acids and other macromolecules [32]. In our current study, all antioxidant activity assays indicated that DOP-40, DOP-60, and DOP-70 possessed strong reduction ability and high scavenging activity of O_2_^−^, hydroxyl and DPPH radicals in a dose-dependent manner. Among them, DOP-70 always showed the strongest antioxidant activity, and the scavenging activities of DOP-40 and DOP-60 were obviously better than that of DOP-50.

Anticancer effects of the four polysaccharide fractions on HepG2 cells were first evaluated using the MTT assay. The results demonstrated that DOP-70 always exhibited higher inhibition rate of HepG2 cells than DOP-40, DOP-50, and DOP-60 at the concentration of 400 μg/ml for 24 h and 48 h. Bax and Bcl-2 are the essential members of Bcl-2 family proteins, which can adjust cell growth and cell death through regulating apoptotic pathway [33]. Furthermore, western blot analysis showed that DOP-40, DOP-60, and DOP-70 decreased the expression level of Bcl-2 and increased the expression level of Bax in HepG2 cells, and DOP-70 had the strongest anticancer effect among them. Above all, the anticancer activities of four polysaccharide fractions were quite similar to their antioxidant effect, and these results also implied that DOP-40, DOP-60, and DOP-70 possessed strong antioxidant and anticancer activities.

## Conclusion

In our current study, four polysaccharide fractions were successfully extracted and purified from *Dendrobium officinale* stems via fractional precipitation with ethanol. The results suggested that DOP-40, DOP-60, and DOP-70 could be developed as promising natural drug therapies or health foods with their antioxidant and anticancer activities.

## Abbreviations

DOP: *Dendrobium officinale* polysaccharides
DMEM: Dulbecco’s modified Eagle’s medium
FBS: fetal bovine serum
MTT: [3-(4, 5-dimethylthiazol-2-yl)-2,5-diphenyl tetrazolium bromide]
MW: molecular weights
HPGPC: high performance gel permeation chromatography
HPLC: high performance liquid chromatography
DPPH: (1,1-diphenyl-2-picryl-hydrazl)
O_2_^−^: superoxide anion radical
wt%: weight percentage
PMSF: phenylmethylsulfonyl fluoride
ECL: enhanced chemiluminescence
