# Correction to: Physicochemical properties of polysaccharides from *Dendrobium officinale* by fractional precipitation and their preliminary antioxidant and anti-HepG2 cells activities in vitro

**DOI:** 10.1186/s13065-018-0471-9

**Published:** 2018-10-11

**Authors:** Shangping Xing, Xiaofeng Zhang, Hannu Ke, Ji Lin, Yuechun Huang, Gang Wei

**Affiliations:** 1 School of Pharmaceutical Science, Guangzhou University of Chinese Medicine, Guangzhou, 510006 China; 2 The First Affiliated Hospital of Guangzhou University of Chinese Medicine, Guangzhou, 510006 China

## Correction to: Chemisty Central Journal (2018) 12:100 10.1186/s13065-018-0468-4

The original version of the article [1] contained a mistake. The

**Fig. 3:**
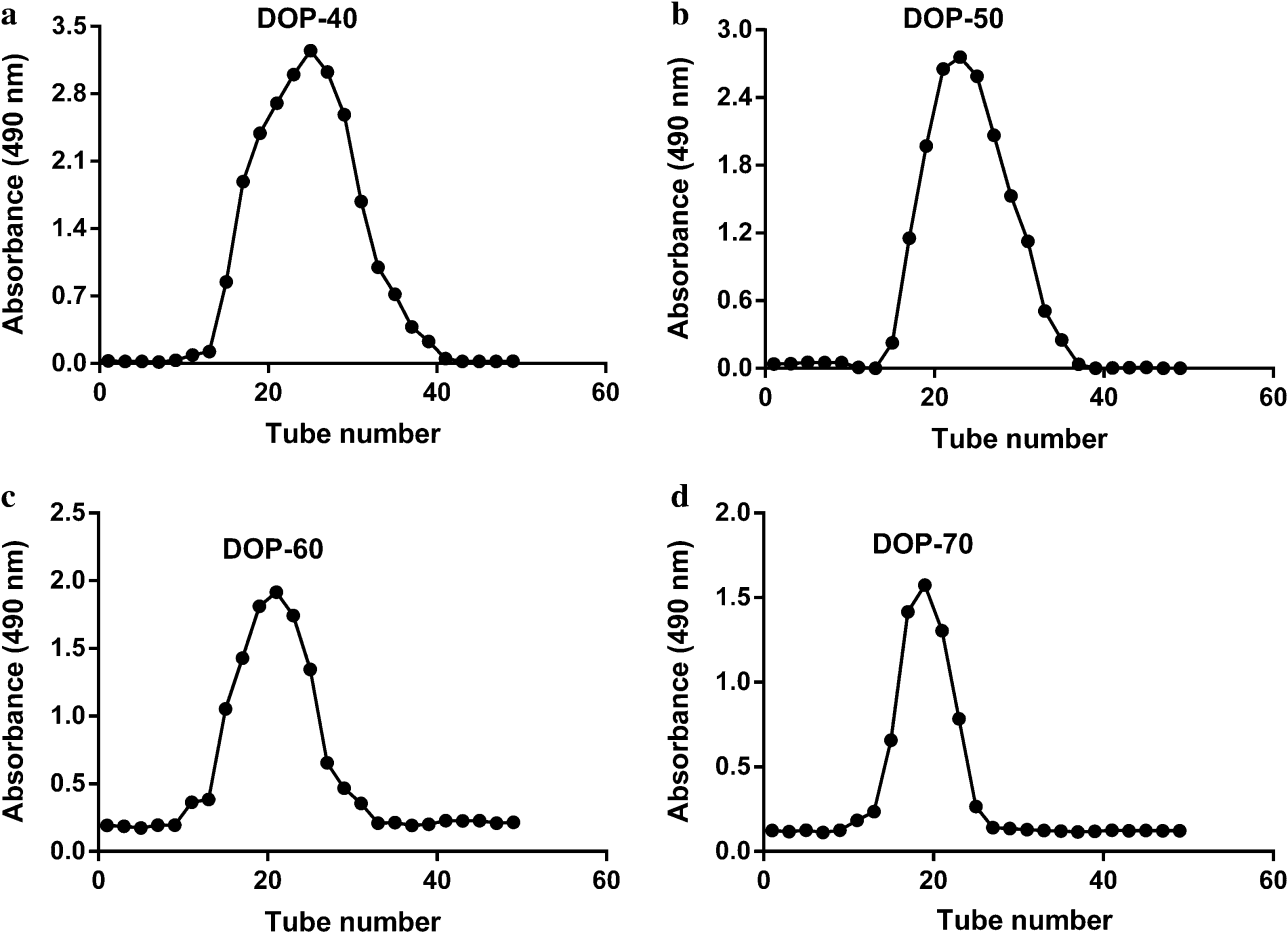
Distilled NaCl elution curves of DOP-40 (**a**), DOP-50 (**b**), DOP-60 (**c**), and DOP-70 (**d**)

Figure legends are right, but the pictures in Figs. 3 and 4 are contrary. The corrected

**Fig. 4:**
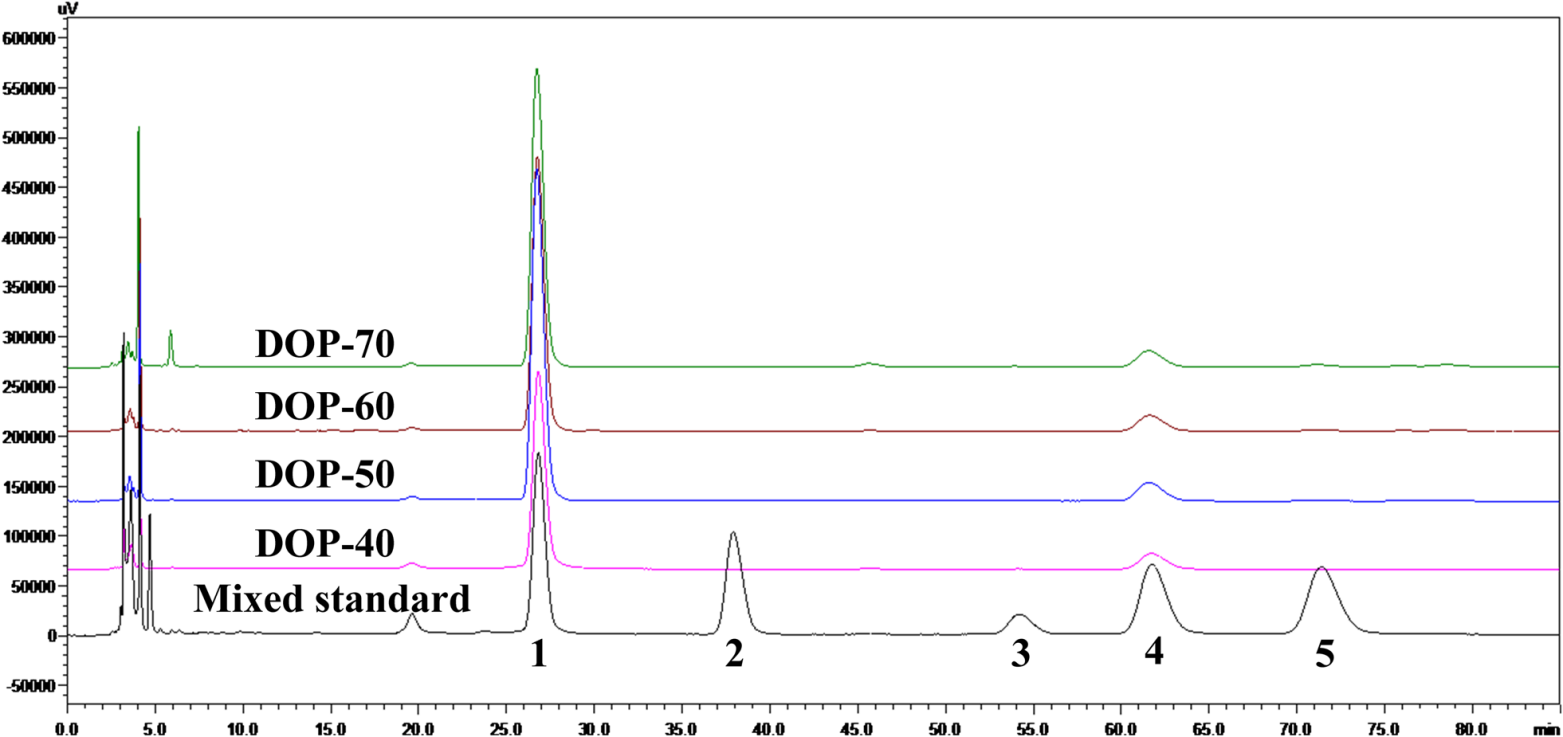
HPLC profile of mixed monosaccharide standards (1-mannose, 2-rhamnose, 3-galactose, 4-glucose, and 5-arabinose), DOP-40, DOP-50, DOP-60, and DOP-70

figures are given below.
